# Systemic Endothelial Function, Plasma Xanthine Oxidoreductase Activity, and Blood Pressure Variability in Patients with Stable Coronary Artery Disease

**DOI:** 10.3390/medicina58101423

**Published:** 2022-10-10

**Authors:** Takashi Hiraga, Yuichi Saito, Kazuya Tateishi, Naoto Mori, Takayo Murase, Takashi Nakamura, Seigo Akari, Kan Saito, Hideki Kitahara, Yoshio Kobayashi

**Affiliations:** 1 Department of Cardiovascular Medicine, Chiba University Graduate School of Medicine, Chiba 260-0856, Japan; 2 Department of Internal Medicine, Chiba Aoba Municipal Hospital, Chiba 260-0852, Japan; 3 Sanwa Kagaku Kenkyusho Co., Ltd., Nagoya 511-0406, Japan

**Keywords:** uric acid, endothelial function, ischemic heart disease, ischemia with no obstructive coronary artery disease

## Abstract

*Background and Objectives:* Although previous studies showed that an activity of xanthine oxidoreductase (XOR), a rate-limiting enzyme in purine metabolism, beyond the serum uric acid level, was associated with the development of coronary artery disease (CAD), the underlying mechanisms are unclear. Because endothelial dysfunction and a greater blood pressure (BP) variability may play a role, we investigated the relations among the endothelial function, XOR, and BP variability. *Materials and Methods:* This was a post-hoc study using pooled data of patients with a stable CAD from two prospective investigations, in which the systemic endothelial function was assessed with the reactive hyperemia index (RHI) and the XOR activity was measured. The BP variability was evaluated using BP measurements during the three- and four-day hospitalization. *Results:* A total of 106 patients with a stable CAD undergoing a percutaneous coronary intervention were included. Of the 106 patients, 46 (43.4%) had a systemic endothelial dysfunction (RHI < 1.67). The multivariable analysis identified a higher body mass index (BMI), female gender, and diabetes as factors associated with an endothelial dysfunction. A higher BMI was also related to an elevated XOR activity, in addition to current smoking. No significant correlation was observed between the RHI and XOR activity. Similarly, the in-hospital BP variability was associated with neither the endothelial function nor XOR. *Conclusions:* Among patients with a stable CAD, several factors were identified as being associated with a systemic endothelial dysfunction or an elevated XOR activity. However, no direct relations between the endothelial function, XOR, and BP variability were found.

## 1. Introduction

Endothelial dysfunction is the earliest stage of a coronary artery disease (CAD), leading to the plaque progression and cardiovascular events [1]. The accumulating evidence has suggested that uric acid, the end-product of purine metabolism in humans, may play an etiologic role in the development of an endothelial dysfunction and coronary plaque progression [2]. In patients with acute coronary syndrome (ACS), an elevated serum uric acid (SUA) level was reportedly associated with an impaired endothelial function and a greater lipid content of coronary plaque [3,4,5]. Previous reports have also indicated a higher SUA level as a maker of future cardiovascular events in patients with ACS and a stable CAD [6,7]. Although a cause-effect relationship has not been established, hyperuricemia may provoke an endothelial dysfunction via the increases in inflammation and oxidative stress, resulting in coronary atherosclerosis and clinical events [2]. Beyond the SUA levels, a recent intracoronary imaging study showed that the elevated activity of xanthine oxidoreductase (XOR), a rate-limiting enzyme in purine metabolism, was associated with coronary lipid-rich plaque in patients with a stable CAD [8]. However, the relation between the endothelial dysfunction and XOR is not fully investigated. An impaired endothelial function is also associated with blood pressure (BP) variability, and elevated SUA levels is known to link to increased fluctuations in the BP [9,10]. Thus, we aimed to evaluate factors associated with an endothelial dysfunction and XOR in patients with a stable CAD and to explore a possible mechanistic impact of BP variability.

## 2. Methods

### 2.1. Study Population

This study was a post-hoc analysis using patient-level pooled data of two prospective studies conducted at Chiba University Hospital [8,11]. A study included 68 patients with a stable CAD undergoing an elective percutaneous coronary intervention (PCI) under near-infrared spectroscopy intravascular ultrasound (NIRS-IVUS) guidance to investigate the relation between the XOR activity and coronary lipid plaque [8]. The other study included 38 patients with a history of a PCI with drug-eluting stent implantation who underwent a coronary angiography to evaluate the coronary endothelial function using a intracoronary low-dose acetylcholine test [11]. Thus, the present dataset included a total of 106 patients with a stable CAD undergoing a PCI. Patients on hemodialysis were excluded. All PCI procedures were carried out per local standard practice [12,13]. In the two studies, a level of the SUA and XOR activity were measured at baseline and the systemic endothelial function was non-invasively evaluated. The studies complied with the Declaration of Helsinki and were approved by the institutional ethics committee at Chiba University Hospital. Written informed consent to participate in the study was obtained by all patients.

### 2.2. Systemic Endothelial Function Assessment

Systemic endothelial function was non-invasively evaluated with the reactive hyperemic index (RHI) using the EndoPAT 2000 device (Itamar Medical Inc., Caesarea, Israel), which is validated as being reproducible and operator-independent [14]. The RHI was measured, as previously reported [15]. Briefly, patients fasted and refrained from taking caffeine, tobacco, and all medications for ≥8 h. The RHI was measured in a quiet and temperature-controlled room in the early morning on the day of or the day after the PCI and coronary angiography. The dedicated probes to measure the arterial pulse waves were placed on the index fingers and a BP cuff was placed on either upper arm. The baseline pulse amplitude was evaluated with the probes for a first 5 min period. The cuff was subsequently inflated for 5 min, and then deflated to induce reactive hyperemia for the next 5 min. The EndoPAT 2000 device automatically calculated the RHI, which is the ratio of amplitude of the signal post-deflation period divided by those at the baseline period, indexed to the contralateral arm. Patients were divided into two groups according to the cut-off value of the RHI of 1.67 [9].

### 2.3. Xanthine Oxidoreductase Evaluation

Blood samples were obtained from an arterial sheath during the PCI and coronary angiography before the injection of unfractionated heparin in the fasting state. The plasma XOR activity was evaluated using a highly sensitive assay using a stable isotope-labeled xanthine and a combination of liquid chromatography and triple quadruple mass spectrometry (Sanwa Kagaku Kenkyusho CO., Ltd., Nagoya, Japan) [16]. The levels of plasma hypoxanthine and xanthine, and the SUA were also measured.

### 2.4. Blood Pressure Variability Assessment

The BP variability during the hospitalization was investigated, as was accomplished in the present studies [17,18]. During the hospitalization for the PCI and coronary angiography, the BP and pulse rate were measured by trained nurses using an automated cuff sphygmomanometer (ES-H55 and ES-H56, Terumo, Tokyo, Japan) at 6:00 AM, 2:00 PM, and 5:00 PM in a routine clinical setting. The PCI and coronary angiography were performed over the course of the four and three days of hospitalization. The BP and PR data were lacking at 6:00 AM on the day of admission and at 2:00 PM and 5:00 PM on the day of discharge. The in-hospital BP variability was evaluated with the standard deviation (SD) and coefficient of variation (CV) of the systolic BP throughout the hospitalization. The CV was defined as the within-subject SD × 100 divided by the average BP level [17,18].

### 2.5. Endpoint and Statistical Analysis

The primary interest of this study was the relation between the endothelial function assessed with the RHI and the XOR activity in patients with a stable CAD. We further investigated the factors associated with a systemic endothelial dysfunction and the XOR activity. As an exploratory analysis, the in-hospital BP variability was also evaluated.

A statistical analysis was performed using SAS software version 9.3 (SAS Institute, Cary, NC, USA). All data are expressed as mean ± SD, median [interquartile range], or frequency (%), as appropriate. The continuous variables were compared with a Student t-test, and the categorical variables were assessed with Fisher’s exact test. The plasma XOR activity was log-transformed because of the skewed distribution. The relation between the variables were analyzed using Pearson’s correlation coefficient. A multivariable analysis using a logistic regression analysis and a multiple linear regression analysis were performed to identify the factors associated with the RHI < 1.67 and XOR activity. The age, sex, and factors associated with the variables in a univariable analysis (*p* < 0.10) were included into the multivariable analysis. The log-transformed XOR and RHI were included into the multivariable models irrespective of the *p* value in the univariable analysis. A *p* value < 0.05 was considered statistically significant.

## 3. Results

**Figure 1:**
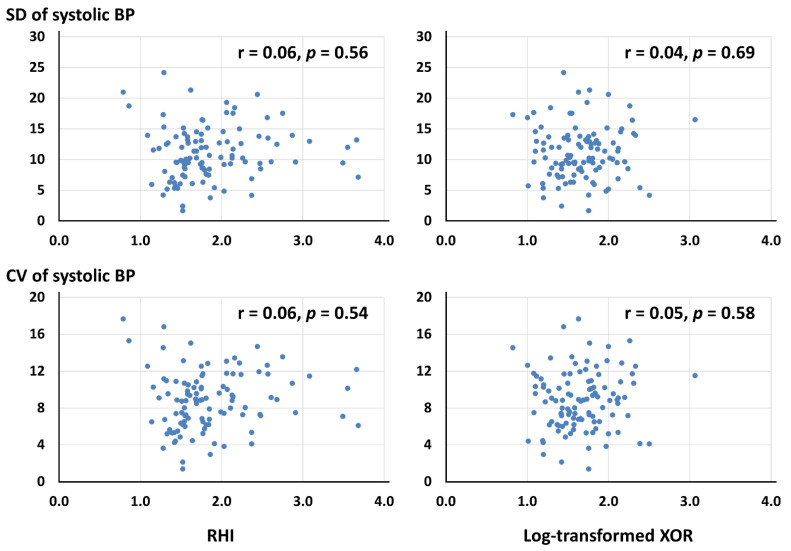
**Relations of the RHI and the log-transformed XOR to the SD and the CV of the systolic BP.** BP: blood pressure, CV: coefficient of variation, RHI: reactive hyperemia index, SD: standard deviation, XOR: xanthine oxidoreductase.

A total of 106 patients with a stable CAD undergoing a PCI were included. The mean SUA level and RHI and median plasma XOR activity were 5.5 ± 1.4 mg/dL, 1.86 ± 0.56, and 43.7 [26.4, 73.4] pmol/h/L, respectively. Of the 106 patients, 46 (43.4%) had a systemic endothelial dysfunction (RHI < 1.67). Table 1 shows the baseline characteristics. The body mass index (BMI) and the prevalence of women and diabetes were higher in patients with the RHI < 1.67 than their counterpart (Table 1). The log-transformed XOR was not significantly correlated with the RHI (r = −0.09, *p* = 0.36). The multivariable analysis identified the female gender, a higher BMI, and the presence of diabetes, as predictors of a systemic endothelial dysfunction (Table 2). A higher BMI was also indicated as a factor associated with a higher log-transformed XOR by the multivariable analysis, in addition to current smoking (Table 3). With respect to the in-hospital BP variability, the BP and pulse rate were measured 6.7 ± 1.3 times during the hospitalization. The mean systolic BP and SD and the CD of the systolic BP were 123.9 ± 12.7 mm Hg, 11.1 ± 4.3, 8.8 ± 3.2, respectively. There were no significant correlations of the SD and CV of the systolic BP to the RHI or the log-transformed XOR (Figure 1).

## 4. Discussion

The present study demonstrated that among patients with a stable CAD undergoing a PCI, an endothelial dysfunction was observed in more than 40%. A higher BMI, the female gender, and diabetes were identified as factors associated with an impaired endothelial function, while XOR was related to a higher BMI and current smoking. There was no direct relationship between the systemic endothelial function and XOR. In addition, neither the endothelial function nor XOR was associated with the in-hospital BP variability.

The dysfunction of the regenerated endothelial cells is recognized as the first step toward coronary atherosclerosis [1]. Numerous previous studies have indicated that an elevated SUA level was associated with the higher likelihood of a CAD, a greater lipid content of coronary plaque, and worse cardiovascular outcomes [4,5,6,7]. The increase in inflammation and oxidative stress and the subsequent endothelial dysfunction during the purine metabolism may play an important role in the development of coronary atherosclerosis in patients with hyperuricemia [2,19]. Additionally, we previously reported that an elevated XOR activity was associated with greater lipid-core plaques beyond the SUA levels [8]. Thus, it is conceivable that an endothelial dysfunction and XOR may be correlated in patients with a CAD.

In the present study, a higher BMI, the female gender, and diabetes were indicated as predictors of a systemic endothelial dysfunction. Among women with no obstructive CAD, the iPOWER study showed that a higher BMI, diabetes, current smoking, and a lower high-density lipoprotein cholesterol level were associated with a lower RHI [20], which is in line with our results. Because another study confirmed the significant relation between the lower RHI and a higher BMI [21], this relationship may be robust. In the multivariable analysis in terms of the XOR activity, a higher BMI was again identified as a significant factor, in addition to smoking. Given that current smoking is also related to an endothelial dysfunction as shown in the iPOWER study [20], the factors associated with the lower RHI and the elevated activity of XOR seem considerably close with each other. However, no direct correlation was found between a lower RHI and an elevated XOR activity in the present study. Therefore, there may be different underlying mechanisms in an endothelial dysfunction and an elevated XOR activity. We believe that the present study provides important mechanistic insights into the association between the endothelial function and purine metabolism and that further studies are warranted to delve into this relation. Because the XOR inhibition is possibly beneficial in preventing cardiovascular events, the therapeutic potential also deserves further evaluation [22].

The BP variability was also evaluated in this study as a possible mechanism of coronary atherosclerosis and worse cardiovascular outcomes in patients with hyperuricemia. The in-hospital BP variability is a recently emerged index of the BP fluctuation, and we and others reported that it was associated with cardiovascular outcomes in patients with a CAD, a peripheral artery disease, and stroke [17,18,23]. Because the BP is routinely measured during hospitalization, in daily practice, the in-hospital BP variability can be readily available. The worse clinical outcomes in patients having a greater BP variability may be, at least partially, explained by the impaired endothelial function [24]. An elevated level of the SUA is reportedly associated with the increased fluctuations in the BP and a non-dipping pattern of the BP [10,25]. However, no significant correlations between the in-hospital BP variability and the RHI or XOR were observed in the present study. Although negative, the results may be important to interpret the clinical studies investigating the BP variability, endothelial function, and purine metabolism. Different mechanisms may play important roles in the development of cardiovascular diseases in patients with a greater BP variability, an impaired endothelial function, and/or an elevated XOR activity.

There are several limitations to our study. This was a single-center, cross-sectional study with a relatively small sample size. Although the data were obtained in a prospective fashion, the present analysis was performed in a post-hoc manner. Although overall similar, the baseline characteristics of two studies were slightly different (Table S1). In the present dataset, the majority were men (i.e., 84%). The systemic endothelial function was evaluated with the RHI in this study, whereas the flow-mediated dilation has been employed in many previous clinical investigations. Even though a meta-analysis indicated that the RHI and the low-mediated dilation have a similar prognostic magnitude [26], a different endothelial function assessment using other modalities may provide different results. In addition, the in-hospital BP variability was used in the present study, but another BP fluctuation metric such as a visit-to-visit BP variability may result in a different outcome.

## 5. Conclusions

Among the patients with a stable CAD undergoing a PCI, a systemic endothelial dysfunction was frequently observed and was associated with a higher BMI, the female gender, and diabetes. While a higher BMI was also identified as a factor related to the elevated activity of XOR, in addition to current smoking, no direct relationship between the endothelial function and XOR was found. Additionally, the in-hospital BP variability was not correlated with the endothelial function and the XOR activity. Future investigations are needed to clarify the underlying mechanisms in the development of cardiovascular disease in patients with an elevated SUA and XOR.

## Figures and Tables

**Table 1:** Baseline characteristics.

Variable	All	RHI ≥ 1.67	RHI < 1.67	*p* Value
	(*n* = 106)	(*n* = 60)	(*n* = 46)	
Age (years)	70.5 ± 10.0	70.9 ± 9.7	69.8 ± 10.6	0.59
Men	89 (84.0%)	55 (91.7%)	34 (73.9%)	0.02
Body mass index (kg/m^2^)	24.5 ± 3.5	23.7 ± 3.0	25.5 ± 4.0	0.01
Hypertension	73 (68.9%)	43 (71.7%)	30 (65.2%)	0.53
Diabetes	48 (45.3%)	18 (30.0%)	30 (65.2%)	<0.001
Dyslipidemia	82 (77.4%)	47 (78.3%)	35 (76.1%)	0.82
Current smoker	15 (14.2%)	8 (13.3%)	7 (15.2%)	0.79
Prior myocardial infarction	29 (27.4%)	16 (26.7%)	13 (28.3%)	1.00
eGFR (mL/min/1.73 m^2^)	70.2 ± 17.0	71.2 ± 15.1	68.8 ± 19.2	0.48
Serum uric acid (mg/dL)	5.5 ± 1.4	5.4 ± 1.3	5.5 ± 1.4	0.67
Log-XOR activity	1.66 ± 0.37	1.67 ± 0.40	1.65 ± 0.35	0.75
Hypoxanthine (μM)	4.40 ± 1.67	4.34 ± 1.72	4.48 ± 1.61	0.67
Xanthine (μM)	0.75 ± 0.97	0.83 ± 1.27	0.64 ± 0.23	0.31
LDL cholesterol (mg/dL)	88.9 ± 24.4	89.7 ± 25.8	87.9 ± 22.7	0.71
HDL cholesterol (mg/dL)	52.3 ± 15.0	54.5 ± 16.5	49.4 ± 12.6	0.08
Non-fasting triglyceride (mg/dL)	145.2 ± 72.4	141.3 ± 74.0	150.2 ± 71.0	0.54
Medical treatment				
ACE-I or ARB	57 (53.8%)	29 (48.3%)	28 (60.9%)	0.24
β-blocker	39 (36.8%)	22 (36.7%)	17 (37.0%)	1.00
Calcium channel blocker	45 (42.5%)	25 (41.7%)	20 (43.5%)	1.00
Diuretic	15 (14.2%)	8 (13.3%)	7 (15.2%)	0.79
Statin	96 (90.6%)	56 (93.3%)	40 (87.0%)	0.32
Oral hypoglycemic agent	40 (37.7%)	14 (23.3%)	26 (56.5%)	<0.001
Reactive hyperemia index	1.86 ± 0.56	2.19 ± 0.51	1.42 ± 0.19	<0.001

ACE-I: angiotensin converting enzyme inhibitor, ARB: angiotensin II receptor blocker, eGFR: estimate glomerular filtration rate, HDL: high density lipoprotein, LDL: low density lipoprotein, RHI: reactive hyperemia index, XOR: xanthine oxidoreductase.

**Table 2:** Factors associated with an endothelial dysfunction (RHI < 1.67).

Variable	Univariable		Multivariable	
	OR (95% CI)	*p* Value	OR (95% CI)	*p* Value
Age (years)	0.99 (0.95–1.03)	0.59	0.98 (0.94–1.03)	0.50
Men	0.26 (0.08–0.80)	0.02	0.19 (0.05–0.73)	0.02
Body mass index (kg/m^2^)	1.17 (1.03–1.32)	0.02	1.19 (1.01–1.39)	0.03
Hypertension	0.74 (0.32–1.69)	0.48		
Diabetes	4.38 (1.93–9.94)	<0.001	4.42 (1.75–11.13)	0.002
Dyslipidemia	0.88 (0.35–2.20)	0.78		
Current smoker	1.17 (0.39–3.49)	0.78		
Prior myocardial infarction	1.08 (0.46–2.56)	0.85		
eGFR (mL/min/1.73 m^2^)	0.99 (0.97–1.02)	0.47		
Serum uric acid (mg/dL)	1.06 (0.80–1.41)	0.67		
Log-XOR activity	0.84 (0.30–2.37)	0.75	0.51 (0.14–1.86)	0.31
Hypoxanthine (μM)	1.05 (0.83–1.33)	0.67		
Xanthine (μM)	0.75 (0.39–1.41)	0.37		
LDL cholesterol (mg/dL)	1.00 (0.98–1.01)	0.71		
HDL cholesterol (mg/dL)	0.98 (0.95–1.00)	0.09	0.99 (0.95–1.02)	0.43
Non-fasting triglyceride (mg/dL)	1.00 (1.00–1.01)	0.53		

CI: confidence interval, eGFR: estimate glomerular filtration rate, HDL: high density lipoprotein, LDL: low density lipoprotein, OR: odds ratio, RHI: reactive hyperemia index, XOR: xanthine oxidoreductase.

**Table 3:** Factors associated with the log-transformed XOR activity.

Variable	Univariable		Multivariable	
	r	*p* Value	β	*p* Value
Age (years)	−0.18	0.07	0.00	0.98
Men	0.18	0.07	0.09	0.36
Body mass index (kg/m^2^)	0.28	0.004	0.24	0.02
Hypertension	0.04	0.71		
Diabetes	0.08	0.40		
Dyslipidemia	0.12	0.21		
Current smoker	0.25	0.008	0.20	0.04
Prior myocardial infarction	0.14	0.16		
eGFR (mL/min/1.73 m^2^)	0.21	0.03	0.13	0.21
Serum uric acid (mg/dL)	0.04	0.65		
Hypoxanthine (μM)	0.05	0.64		
Xanthine (μM)	−0.15	0.12		
LDL cholesterol (mg/dL)	0.02	0.88		
HDL cholesterol (mg/dL)	−0.02	0.80		
Non-fasting triglyceride (mg/dL)	0.11	0.27		
RHI	−0.09	0.36	0.00	0.98

eGFR: estimate glomerular filtration rate, HDL: high density lipoprotein, LDL: low density lipoprotein, RHI: reactive hyperemia index, XOR: xanthine oxidoreductase.

## Data Availability

Not applicable.

## Supplementary Materials

The following supporting information can be downloaded at: https://www.mdpi.com/article/10.3390/medicina58101423/s1, Table S1: Baseline characteristics between the 2 original studies.
