# Supplementary material for: Systemic Endothelial Function, Plasma Xanthine Oxidoreductase Activity, and Blood Pressure Variability in Patients with Stable Coronary Artery Disease
Source: Medicina (Kaunas). 2022 Oct 10;58(10):1423. doi: 10.3390/medicina58101423 (PMC9611040; doi:10.3390/medicina58101423)
Supplement: Supplementary file 1 [file medicina-58-01423-s001.zip › Table S1.pdf]

Table S1. Baseline characteristics between the 2 original studies

Variable	Study #1 (n=68)	Study #2 (n=38)	p value
Age (years)	70.3±10.8	70.7±8.7	0.86
Men	58 (85.3%)	31 (81.6%)	0.78
Body mass index (kg/m^2)	24.3±3.8	24.8±3.1	0.55
Hypertension	46 (67.7%)	27 (71.1%)	0.83
Diabetes	31 (45.6%)	17 (44.7%)	1.00
Dyslipidemia	48 (70.6%)	34 (89.5%)	0.03
Current smoker	12 (17.7%)	3 (7.9%)	0.25
Prior myocardial infarction	18 (26.5%)	11 (29.0%)	0.83
eGFR (ml/min/1.73 m^2)	71.5±17.8	67.8±15.4	0.29
Serum uric acid (mg/dl)	5.5±1.5	5.4±1.2	0.51
Log-XOR activity	1.65±0.40	1.68±0.33	0.72
Hypoxanthine (μM)	4.32±1.68	4.52±1.66	0.55
Xanthine (μM)	0.56±0.24	1.08±1.55	0.007
LDL cholesterol (mg/dl)	92.4±26.3	82.7±19.5	0.050
HDL cholesterol (mg/dl)	51.9±16.4	53.0±12.4	0.73
Non-fasting triglyceride (mg/dl)	139.6±65.5	155.2±83.5	0.29
Medical treatment			
ACE-I or ARB	33 (48.5%)	24 (63.2%)	0.16
β-blocker	27 (39.7%)	12 (31.6%)	0.53
Calcium channel blocker	31 (45.6%)	14 (36.8%)	0.42
Diuretic	9 (13.2%)	6 (15.8%)	0.77
Statin	58 (85.3%)	38 (100%)	0.01
Oral hypoglycemic agent	28 (41.2%)	12 (31.6%)	0.41
Reactive hyperemia index	1.86±0.52	1.85±0.63	0.89

ACE-I angiotensin converting enzyme inhibitor; *ARB* angiotensin II receptor blocker; *eGFR* estimate glomerular filtration rate; *HDL* high density lipoprotein; *LDL* low density lipoprotein; *RHI* reactive hyperemia index; *XOR* xanthine oxidoreductase.
